# Enhancing the Electrochemical Activity of 2D Materials Edges through Oriented Electric Fields

**DOI:** 10.1021/acsnano.4c06341

**Published:** 2024-07-16

**Authors:** Hao Wang, Ding-Rui Chen, You-Chen Lin, Po-Han Lin, Jui-Teng Chang, Jeyavelan Muthu, Mario Hofmann, Ya-Ping Hsieh

**Affiliations:** † Institute of Atomic and Molecular Sciences, Academia Sinica, Taipei 10617, Taiwan; ‡ Department of Physics, National Taiwan University, Taipei 10617, Taiwan; § International Graduate Program of Molecular Science and Technology, National Taiwan University, Taipei 10617, Taiwan; ∥ Molecular Science and Technology Program, Taiwan International Graduate Program, Academia Sinica, Taipei 10617, Taiwan; ⊥ Department of Low Dimensional Systems, J. Heyrovský Institute of Physical Chemistry, Prague 18200, Czech Republic

**Keywords:** 2D materials, 2D edges, electrochemistry, oriented electric fields, HER

## Abstract

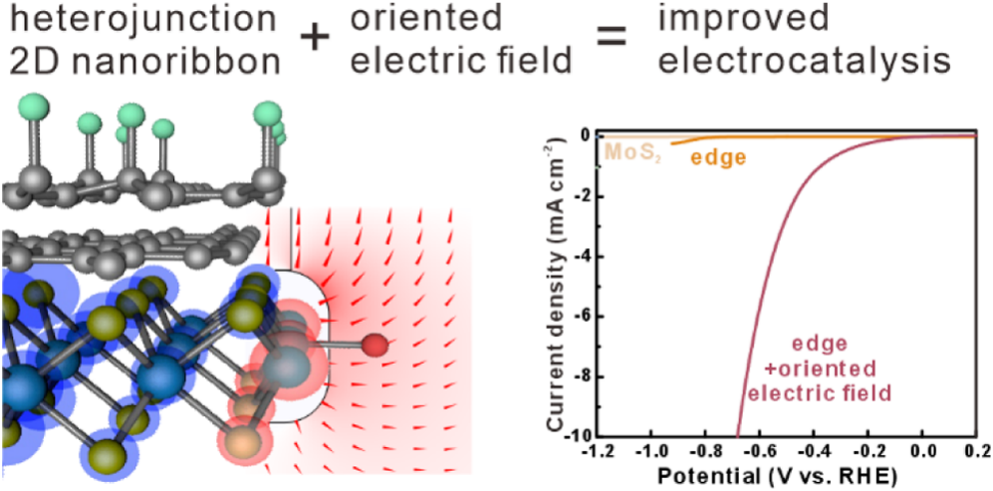

The edges of 2D materials have emerged as promising electrochemical catalyst systems, yet their performance still lags behind that of noble metals. Here, we demonstrate the potential of oriented electric fields (OEFs) to enhance the electrochemical activity of 2D materials edges. By atomically engineering the edge of a fluorographene/graphene/MoS_2_ heterojunction nanoribbon, strong and localized OEFs were realized as confirmed by simulations and spatially resolved spectroscopy. The observed fringing OEF results in an enhancement of the heterogeneous charge transfer rate between the edge and the electrolyte by 2 orders of magnitude according to impedance spectroscopy. Ab initio calculations indicate a field-induced decrease in the reactant adsorption energy as the origin of this improvement. We apply the OEF-enhanced edge reactivity to hydrogen evolution reactions (HER) and observe a significantly enhanced electrochemical performance, as evidenced by a 30% decrease in Tafel slope and a 3-fold enhanced turnover frequency. Our findings demonstrate the potential of OEFs for tailoring the catalytic properties of 2D material edges toward future complex reactions.

## 1 Introduction

Electrochemical reactions are at the heart of efforts to convert sustainable energies into needed products, such as fuels and chemicals.^1^ Achieving the necessary scale to make a global impact requires noble metal-free catalysts with high efficiency and low cost.^2,3^ 2D materials edges have demonstrated great potential in electrocatalysis due to their high surface area, large tunability of chemical character, and superior catalytic activity.^4,5^ Despite significant research, however, the activity of 2D material edges remains below that of noble metal catalysts.^6^

A promising route to enhance the catalytic performance of 2D materials could be oriented electric fields (OEFs).^7,8^ Through electrostatic modification of the bond alignment between catalyst and reactant, the efficiency and selectivity of chemical reactions can be enhanced.^9^ Oriented external electric fields have been reported to enhance oxygen evolution reaction,^10^ organic reactions,^11^ carbon nanotube growth,^12^ and carbon dioxide activation.^13^

OEFs seem to be particularly well suited for hydrogen evolution reactions (HER) due to the importance of properly oriented bonds in the proton transfer process.^14^ OEF-induced variations of the homogeneous charge distribution within the double layer^15^ could lower the energetic cost of reorienting individual protons within the collective dipole of the double layer and decrease the energetic barrier of reactant motion.^14^

Recent findings on 2D materials subjected to external field modulation^16,17^ and defect-assisted internal field engineering^18,19^ indicate the potential impact of OEFs on the electrochemical reactivity of 2D materials. Unfortunately, the described approaches are only able to enhance the reactivity of a 2D material’s basal plane and not its edge. Therefore, the observed enhancements have been limited by the low initial electrocatalytic activity of the basal plane compared to 2D materials edges.^20^

We here demonstrate the realization of OEFs at the 2D material edge and its impact on the electrochemical performance. Through the design of a vertical heterojunction, a permanent internal electric dipole can be produced. A bottom-up patterning process was utilized to convert the 2D heterojunction into nanometer-wide ribbon arrays whose electrostatics are dominated by fringing fields at their edges. Optical and impedance spectroscopic characterization at the vertical MoS_2_/graphene/fluorographene edge confirms the formation of a sizable in-plane dipole that enhances the heterogeneous charge transfer reaction by 2 orders of magnitude. The presented OEF-enhanced 2D edge reactivity was applied to hydrogen evolution reactions (HER), where ab initio calculations suggest a field-induced decrease in the hydrogen adsorption energy. Experimental HER confirms the impact of OEFs on MoS_2_ edge reactivity and yields a 30% decrease in Tafel slope compared to pristine edges and a 3-fold increase in turnover frequency. Our results demonstrate the impact of tailoring the catalytic activity of 2D materials for the electrocatalytic and photocatalytic generation and storage of energy.

## 2 Results

**Figure 1:**
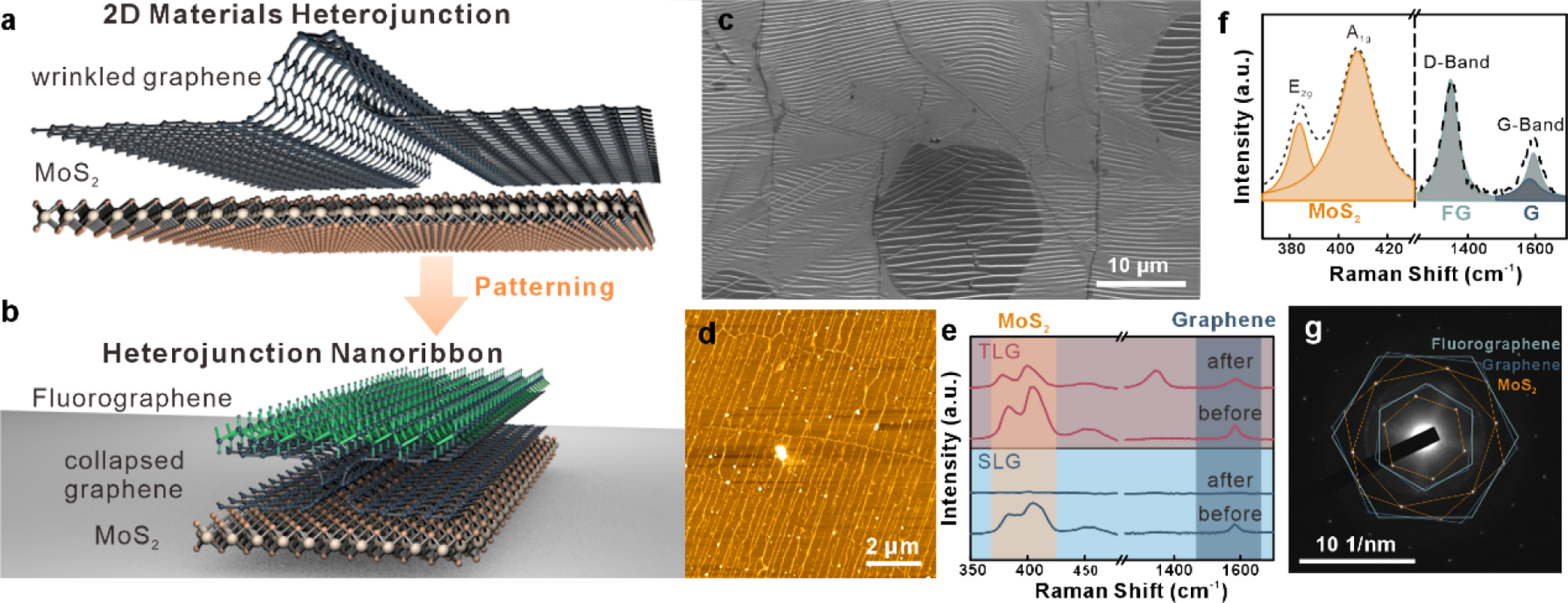
Formation of 2D heterojunction nanoribbons: (a) Schematic illustration of he thickness-dependent pattern transfer process. (b) Resulting MoS_2_/graphene/fluorographene heterojunction nanoribbon. (c) Scanning electron micrograph of CVD-grown graphene on copper foil, showing a large array of wrinkles due to the thermal expansion difference between graphene and copper foil. (d) Atomic force micrograph of heterostructure nanoribbon array. (e) Raman spectra before and after patterning, indicating removal of SLG regions and retention of TLG regions. (f) Deconvolution of Raman features into contributions from MoS_2_, graphene, and fluorographene. (g) Selected area diffraction pattern of heterojunction nanoribbon with the assignment of all three components.

To produce OEFs, we utilize the permanent dipole that is produced between materials of different work functions. Such internal electric fields can be produced in van der Waals assemblies of different 2D materials and have demonstrated internal field strengths in excess of 1 V nm^–1^.^21^ We realize such vertical 2D heterojunctions by sequential transfer of CVD-grown MoS_2_ and graphene onto a SiO_2_/Si substrate (Figure 1a; more details in the Supporting Information). These structures, however, exhibit OEFs that are limited to the out-of-plane direction and are not compatible with the envisioned application to 2D edges. Moreover, previous work has indicated that edge-dominated electrochemical characteristics only occur if carrier transport is confined to 1D nanoribbons with nanometer widths.^22^

We address the vision of combining 2D heterojunction-based OEFs and edge-dominated electrochemistry by developing heterojunction nanoribbons, where vertical vdW stacks are confined into 1D. These complex nanostructures were realized through a self-aligned bottom-up patterning method (Figure 1b). Previous research demonstrated the achievable atomic precision^23^ and high throughput^22^ of thickness-dependent graphene pattern transfer method: Through a self-stabilization process, fluorination of graphene would selectively remove single-layer regions and retain multilayers.^23^

The patterning method was employed to convert the vertical 2D heterojunctions into nanoribbons by exploiting the graphene wrinkles as templates. Graphene wrinkles of nanometer width and high aspect ratio form naturally due to the mismatch in thermal expansion coefficient between the copper growth substrate and graphene during cooling from growth conditions. Scanning electron microscopy confirms the formation of large arrays of parallel wrinkles after CVD growth. Upon transfer from their growth substrate, capillary forces collapse these wrinkles into trilayer graphene (TLG) nanoribbons within the single-layer graphene (SLG) (Figure 1c).^23^

The thickness-dependent pattern transfer process will selectively remove the SLG while the TLG serves as a hard mask for the underlying 2D materials. Indeed, atomic force microscopy confirms the formation of heterojunction nanoribbon arrays with high density and parallel alignment with the copper substrate’s crystalline texture^24^ (Figure 1d). The removal of excess MoS_2_ not covered by the TLG is demonstrated by Raman spectroscopy of the characteristic E_2g_ and A_1g_ modes at 384 and 407 cm^–1^, respectively (Figure 1e).

We further employed Raman spectroscopy to assess the components of the heterojunction. Deconvolution of Raman spectra demonstrates the collocation of the MoS_2_ features and the graphene G-Band as expected. The occurrence of a pronounced D-Band and a broad G-Band feature further indicates the transformation of the top graphene layer into fluorographene, in agreement with previous findings (Figure 1f).^23^ This patterning process is further corroborated by selected area diffraction, which shows the characteristic patterns for MoS_2_, graphene, and fluorographene (Figures 1g and S3). The misalignment between the lattices is expected due to the random orientation of the components during transfer and wrinkle collapse.

**Figure 2:**
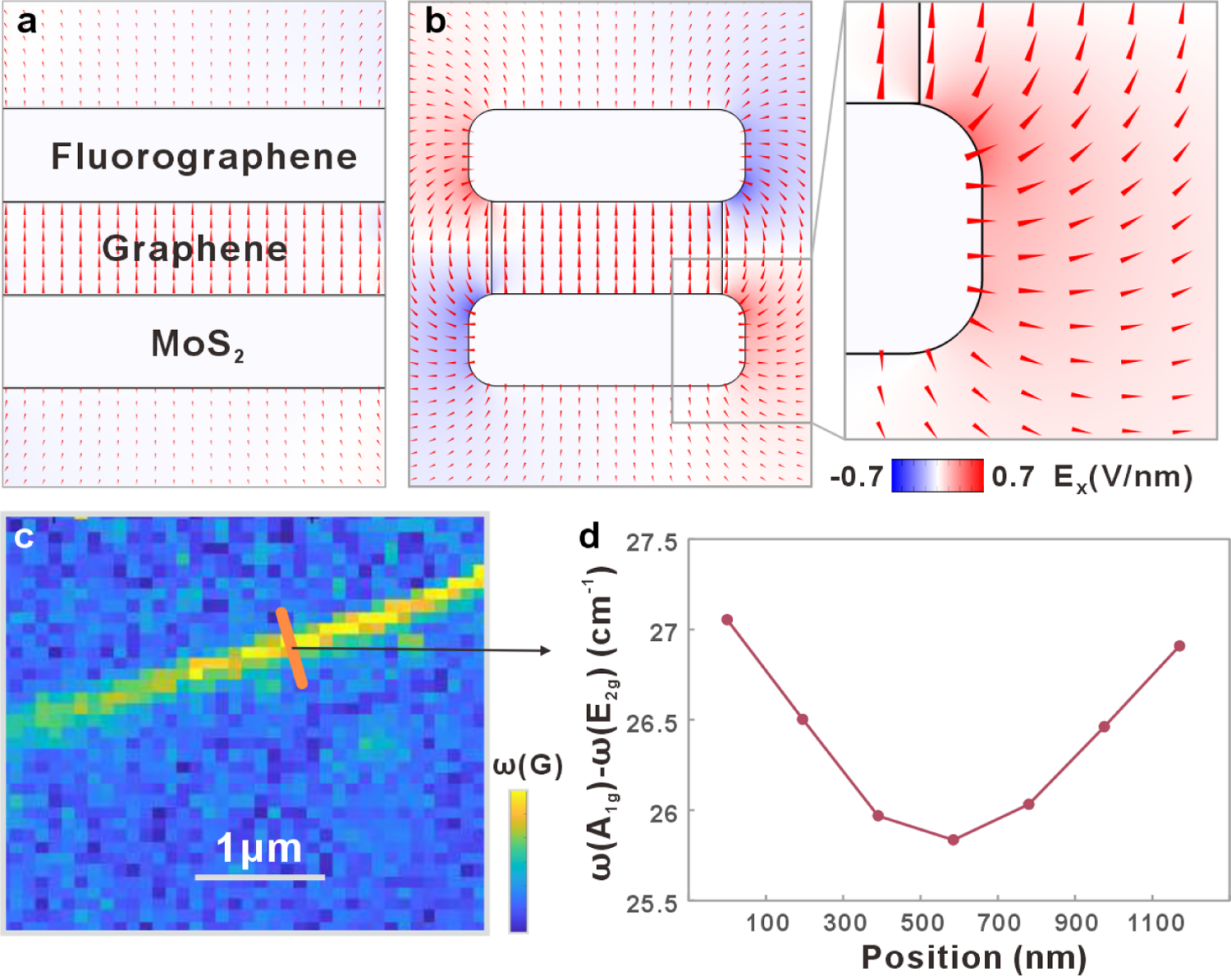
OEFs in heterojunction nanoribbons: Finite element simulation of electric fields in MoS_2_/graphene/fluorographene heterojunction (a) for a laterally extended 2D vertical heterojunction; (b) for a heterojunction nanoribbon. (c) Raman mapping of G-Band intensity of a single nanoribbon. (d) Raman peak shift between E_2g_ and A_1g_ across the nanoribbon, as indicated in panel (c).

The presented approach produces a complex ternary vertical heterojunction in ultranarrow ribbons. The confinement to nanometer width (see Figure S4 for statistical characterization) imparts the heterostructure with edge-dominated electrostatics: whereas extended vertical 2D heterojunctions will exhibit a one-dimensional field distribution, the discontinuous dielectric environment and the absence of charge screening is expected to yield a complex electric field pattern.^25^ To confirm this hypothesis, we conducted finite element simulations of the electrostatics utilizing the experimentally identified heterojunction composition. A work function difference of 0.45 eV between MoS_2_^26^ and fluorographene^27^ was assumed. The simulation results confirm a homogeneous field emerging in the extended heterojunction (Figure 2a). The edge-dominated heterojunction, on the other hand, exhibits a complex field distribution with a pronounced fringing field at the edges (Figure 2b). The resulting OEF is almost parallel to the nanoribbon edge and, thus, penetrates the edge/electrolyte interface. The strength of these fringing OEF reaches up to 0.7 V nm^–1^, which is comparable to the built-in field of vertical heterojunctions.

To experimentally confirm this prediction, we conducted spectroscopic characterization on an individual heterojunction nanoribbon. Spatially resolved Raman spectroscopy demonstrates the variation of Raman features within the ribbon (Figure 2c). We identify the presence of an OEF at the MoS_2_ edge by spatially mapping the difference in Raman shift between the A_1g_ and E_2g_ features due to their established sensitivity to electrostatic effects.^28^ We observe a clear upshift in the characteristic peak difference between the ribbon center and edges that correspond to an enhanced hole accumulation at the edges (Figure 2d). A similar trend can also be observed for the graphene G-band position, corroborating the formation of a lateral dipole that is pointing toward the nanoribbon edge (Figure S5).

**Figure 3:**
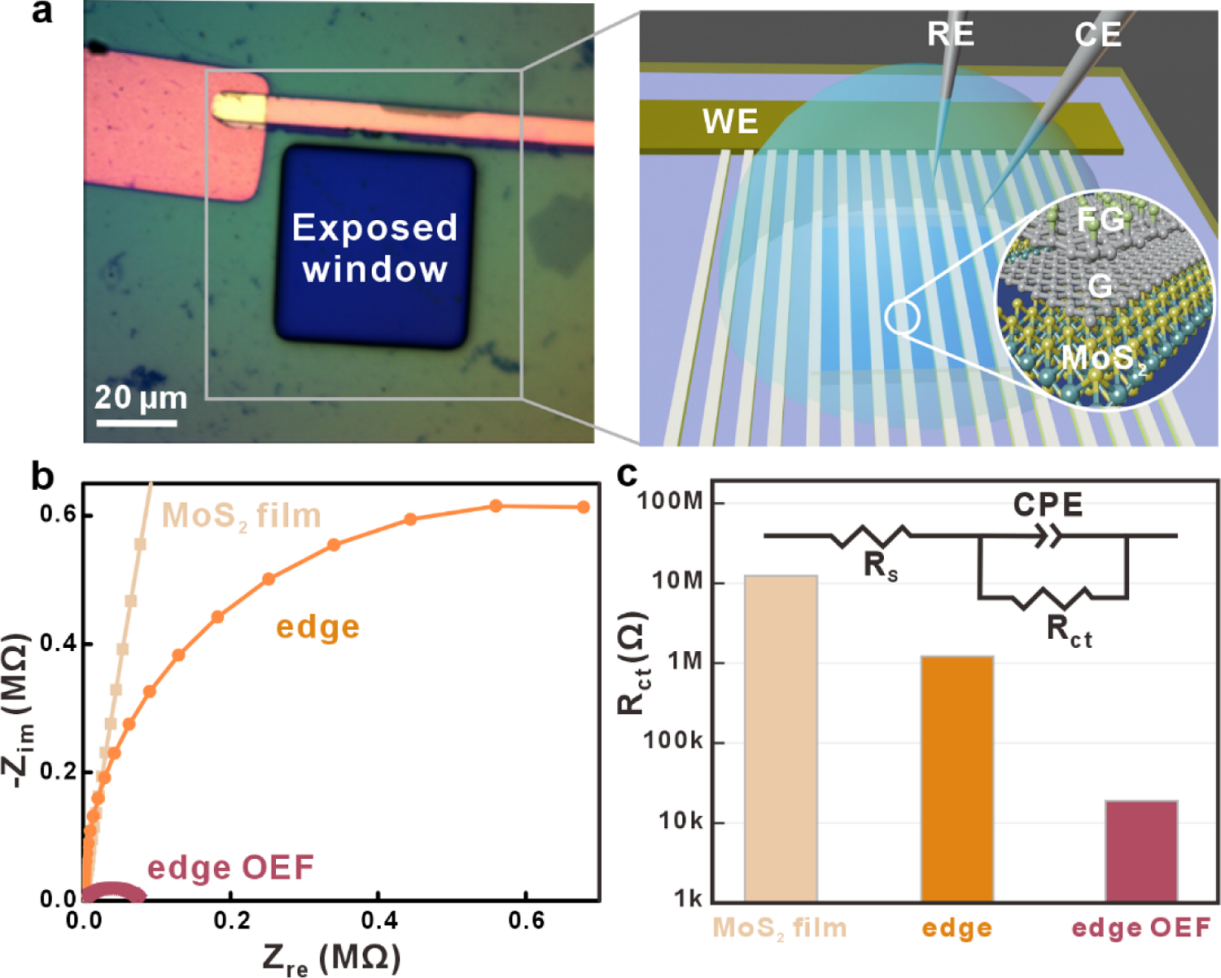
Electrochemical performance of OEF-enhanced edges. (a) Optical micrograph and schematic illustration of heterojunction nanoribbon array within a reaction window and gold electrode for electrochemical measurement. (b) Nyquist impedances and (c) charge transfer resistance of the MoS_2_ flake, MoS_2_ nanoribbon, and heterojunction nanoribbon.

We evaluate the impact of the observed fringing of the OEF on the electrochemical performance of MoS_2_ edges. For this purpose, we fabricate a microelectrode cell that permits contact with multiple nanoribbons in parallel (Figure 3a). Photolithography was utilized to open a window in a photoresist layer that protects the metal electrode and exposes only a 50 μm × 50 μm large area of the nanoribbon array.

Electrochemical impedance spectroscopy (EIS) was conducted to assess the heterogeneous charge transfer kinetics at the edge (Figure 3b). The observed large semicircle in the Nyquist plot with negligible separation from the origin suggests that the electrochemical reaction is limited by a heterogeneous charge transfer step between the electrode and the electrolyte.^29^

Fits to an equivalent circuit permit quantification of this observation. We approximate the structure with an electrolyte resistance and a series RC circuit, which represents the charge transfer at the edge/electrolyte interface (inset, Figure 3c). A decrease in charge transfer resistance by 1 order of magnitude is observed as the continuous film is patterned into a nanoribbon array, in agreement with previous reports.^22^ However, this enhancement is surpassed by edge OEF, and the heterogeneous charge transfer resistance decreases by 2 orders of magnitude compared to pristine edges.

**Figure 4:**
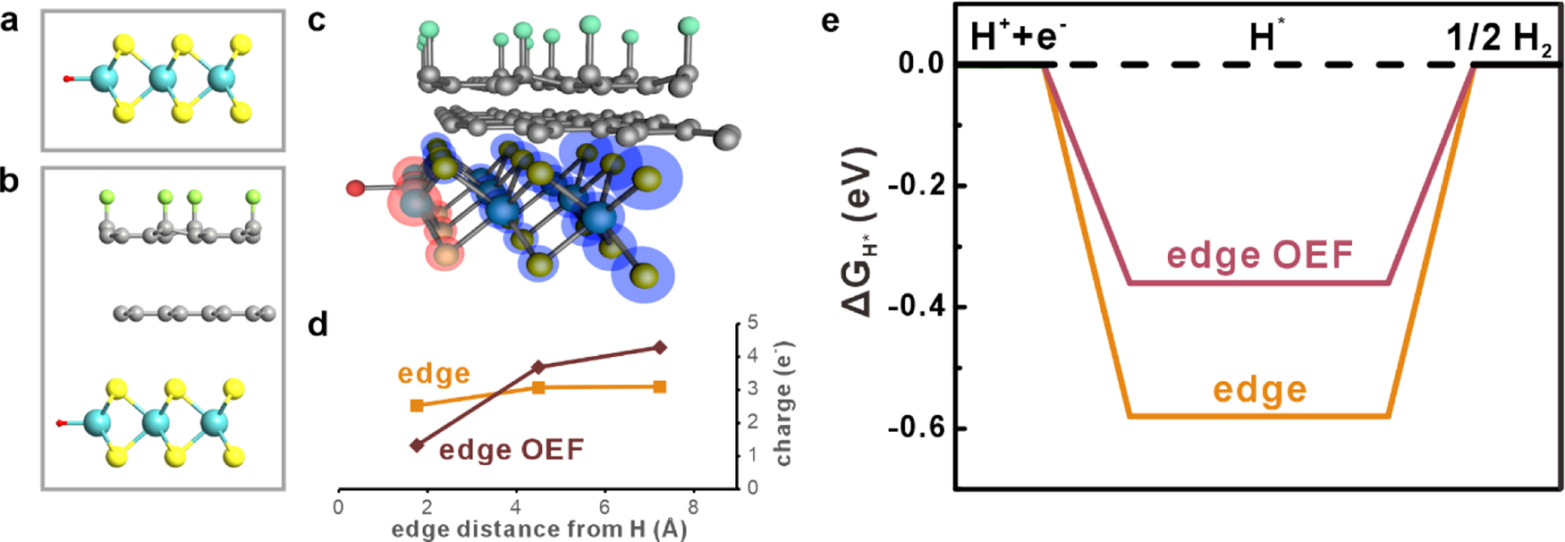
Ab initio simulation of edge-enhanced OEF. (a) Side view of supercell model of MoS_2_ nanoribbon and (b) heterojunction nanoribbon bonded with a hydrogen atom. Elements: blue, Mo; yellow, S; green, F; and red, H. (c) Difference in Bader charge between the MoS_2_ nanoribbon and nanoribbon heterostructure. The blue and red spheres are proportional to the charge accumulation and depletion, respectively. (d) Bader charge analysis for rows of Mo atoms parallel to the edge of the MoS_2_ nanoribbon in the pristine and OEF case. (e) Gibbs free energy Δ*G*_H_* vs reaction coordinates for hydrogen adsorption on Mo edge for pristine and heterojunction conditions.

To understand the OEF-enhancement mechanism on 2D materials edges, we conduct ab initio simulations on the hydrogen evolution reaction due to its conceptual simplicity and impact on sustainable energy generation. The pristine MoS_2_ edge was compared to the OEF-enhanced edge within the nanoribbon heterojunction composed of MoS_2_/graphene/fluorographene (Figure 4a,b). We focus on the Mo-rich edge of MoS_2_ due to its proven electrochemical activity for hydrogen evolution reactions (more details in Figures S6 and S7).^30,31^

Adsorption of a hydrogen atom results in a charge redistribution within MoS_2_ due to the difference in electron affinity (Figure 4c). When investigating the charge distribution at each row of Mo atoms within the nanoribbon, the impact of the edge OEF can be seen. Compared with the near-constant charge distribution within a pristine nanoribbon, the edge-OEF shows a decrease in charge close to the adsorbed hydrogen (Figure 4d). The Mo atom neighboring the proton lowers its charge by 0.47e^–^, suggesting a significant effect of the OEFs on the interaction between reactants.

To quantify the impact of the OEFs on the HER, we investigate the Gibbs free energy of hydrogen adsorption Δ*G*_H_*, as it is considered the rate-limiting step for the reaction. A negative value of Δ*G*_H_* denotes a strong bond, which is challenging for an H atom to break during desorption, while a positive value represents a weakly bound H atom that limits adsorption. Consequently, a Δ*G*_H_* value close to 0 represents the optimal balance between adsorption and desorption. Pristine MoS_2_ edges exhibit a Δ*G*_H_* of −0.58 eV, which agrees with previous reports^31^ and indicates a desorption-limited reaction process. The charge redistribution by the OEFs simplifies the desorption, and a Δ*G*_H_* of −0.36 eV is calculated (Figure 4e). While the employed PBE functional is known to overestimate the absolute value of the hydrogen adsorption energy,^32,33^ the observed trend toward the optimal adsorption energy corroborates the impact of edge OEFs on electrochemical reactions.

**Figure 5:**
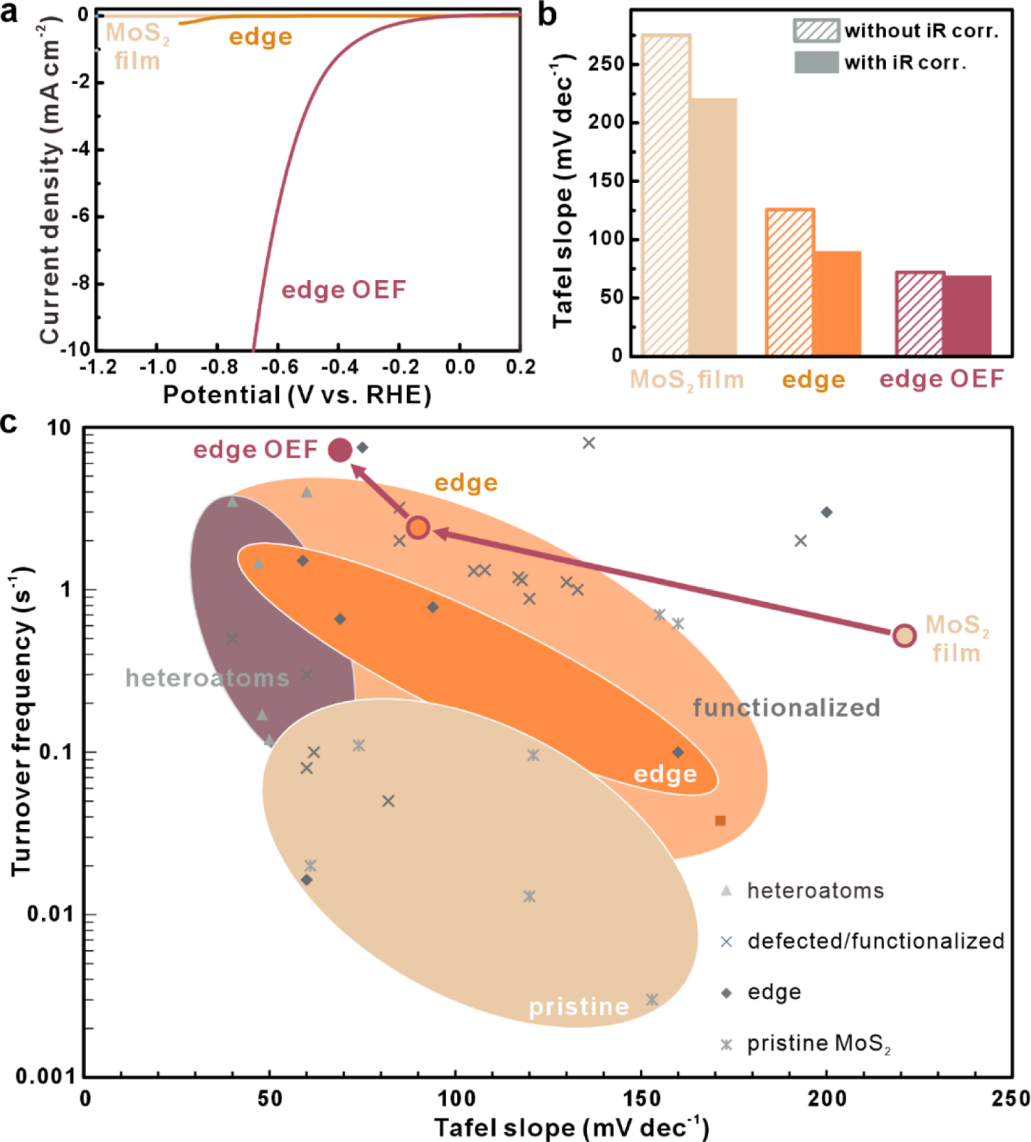
Electrochemical HER performance of OEF-enhanced edges. (a) Polarization curves MoS_2_ flake, MoS_2_ nanoribbon, and heterojunction nanoribbon (more details on the current density calculation are provided in the Supporting Information). (b) Tafel slopes with and without *iR* correction. (c) Comparison of literature values of Tafel slope versus TOF to our results (more details, including a comparison of reaction current density, are provided in the Supporting Information).

We confirm these predictions experimentally by conducting HER. We first measure the typical polarization curve in acidic solution (Figure 5a). To permit comparison, the reaction current at a given potential was normalized by the basal plane area, as detailed in the Supporting Information. As predicted, the MoS_2_ flake exhibited the poorest HER performance in agreement with the EIS results, confirming the limited catalytic activity of the MoS_2_ basal plane.^34^ The formation of edges enhances the HER performance, as expected. The importance of the use of the OEFs is demonstrated by the significant increase in exchange current density in the HER polarogram (Figure 5a).

The extracted reaction current density represents the lower boundary, as it underestimates the contribution of the edge, but the extracted value is among the highest reported values for 2D materials-based HER catalysts, as detailed in the Supporting Information (Table S3). Future efforts could further enhance the performance by increasing the density of nanoribbons within the array.^22^

We quantify the kinetics of the HER process by extracting the Tafel slope (Figure 5b). Recent work has demonstrated that this feature not only allows the evaluation of the rate-determining step during HER but also provides information about the material’s properties: analysis of the uncompensated resistance reveals restrictions to carrier transport within the electrode.^6^ We find that the Tafel slope after *iR* correction is reduced (more details are provided in the Supporting Information), indicating the importance of accounting for the potential drop across the nanoribbon. The reduction was more substantial for the pristine nanoribbon than for the OEF-enhanced nanoribbon, which suggests the synergy of the components in enhancing the conduction. The small impact on the electrochemical parameters, however, further confirms the controlling effect of the OEFs over other heterojunction parameters. The corrected Tafel slope approaches 69 mV dec^–1^, and a sample-to-sample variability of 7% was observed.

Finally, the turnover frequency is a quantitative measure of how quickly a catalyst can facilitate a specific reaction per unit time, following the methodology outlined in a previous study by Jaramillo et al.^35^ (more details are provided in the Supporting Information). We observe a tripling in TOF between pristine and heterostructure edges, with the highest TOF reaching 7.23 s^–1^. The combination of low Tafel slope and high turnover frequency demonstrates the impact of OEFs toward HER (Figure 5c, see Table S3 for a comparison to references). Future work could further enhance this performance by utilizing heterojunction components with larger differences in work function.

## 3 Conclusions

We have demonstrated the OEF-induced enhancement of 2D material edge-based electrochemistry. Through a powerful and universal bottom-up patterning approach, heterojunction nanoribbons were produced that exhibit a complex ternary composition and nanometer width. The resulting edge-dominated electrochemical characteristics exhibit significant differences in electrochemical performance compared with bare nanoribbons. Spectroscopic characterization and ab initio simulations demonstrate the formation of an oriented electric field that modifies the charge transfer dynamics at the edge/electrolyte interface. The advantage of our universally applicable edge OEF approach was demonstrated by a 30% decrease in Tafel slope and superior turnover frequency over previous 2D materials-based HER catalysts. Our results demonstrate the impact of OEFs toward enhancing the reactivity of 2D material catalysts.

## 4 Experimental Section

### 4.1 Material Preparation

#### 4.1.1 Synthesis of Graphene

Copper foil (Alfa Aesar 46365, purity 99.8%) was used as the catalyst for CVD-graphene growth. Adhering to previously reported methodology, the copper foil was stackied between graphite foil in a 1 in. quartz tube and first annealed at 1020 °C for 70 min under 10 sccm H_2_. The growth of graphene was initiated by introducing a gas mixture comprising 200 sccm H_2_ and 10 sccm CH_4_ at 1020 °C for 6 h. Subsequently, the sample was gradually cooled to room temperature at a rate of 10 °C min^–1^ under 10 sccm of H_2_.

#### 4.1.2 Synthesis of MoS_2_

We use chemical vapor deposition (CVD) to synthesize a uniform MoS_2_ film. Initially, a Si substrate with a 300 nm SiO_2_ film that serves as the growth substrate was cleaned by 5 min sonication in acetone, followed by a 10 min oxygen plasma treatment. Subsequently, a spin-coated solution of sodium chloride (0.01 g mL^–1^ NaCl and 2.5 × 10^–4^ M NaOH) was applied to the substrate to facilitate growth. Graphite foil with 40 nm molybdenum trioxide (MoO_3_) deposited by e-beam evaporation served as the molybdenum (Mo) precursor and was oriented face down toward the substrates. This assembly was placed inside the 3 in. quartz tube in the center of the furnace.

MoS_2_ growth followed a two-step heating process. All of the processes were conducted under 350 sccm H_2_S (1% H_2_S + 99% Ar). First, the temperature was ramped up to 700 °C in 20 min. Second, the temperature was further increased from 700 to 900 °C within an additional 20 min. Then, the growth temperature was held at 900 °C for 10 min. Following this two-step heating process, the samples underwent a gradual cooling process to reach room temperature.

### 4.2 Patterning of Heterojunction Nanoribbon and MoS_2_ Nanoribbon

For MoS_2_/graphene/fluorographene heterojunction nanoribbon patterning, graphene was transferred onto Si/SiO_2_/MoS_2_ substrates through a well-established wet chemical process.^36^ Subsequently, a 25 W CF_4_ corona discharge plasma was employed to pattern both multilayer graphene and graphene wrinkles under a pressure of 600 mTorr for 10 min until the characteristic Raman signal of the monolayer graphene region vanishes.

For MoS_2_ nanoribbon patterning, we removed the graphene/fluorographene part of the heterojunction through 40 s of oxygen plasma treatment.

### 4.3 Characterization

Atomic force microscopy (AFM) and scanning electron microscopy (SEM) data were collected in a Bruker Dimension Icon and FESEM Nova 450.

Raman spectroscopy and mapping were performed in a home-built micro-Raman system with 532 nm excitation. The advanced characterization and ribbon width definition were carried out by high-resolution transmission electron microscopy (JEM2100F).

### 4.4 Electrochemical Measurement

Electrochemical characterization was conducted using a three-electrode system on a CHI electrochemical station. Platinum (Pt) and silver/silver chloride (Ag/AgCl) electrodes served as the counter and reference electrodes, respectively. The prefabricated gold electrode on the sample acted as contact to the working electrode covered with photoresist to ensure that all contributions to the hydrogen evolution reaction (HER) originated from the exposed window. The current density normalization procedure is detailed in the Supporting Information.

Polarization curves were recorded in a 0.5 M H_2_SO_4_ solution with a scan rate of 0.07 V s^–1^. The applied voltage spanned from 0 to −2 V. All of the reported potentials in our study were referenced to the reversible hydrogen electrode (RHE), determined by the equation
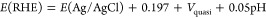
1Electrochemical impedance spectroscopy (EIS) was carried out over a frequency range spanning 1 Hz to 1 MHz. The amplitude used was 0.5 V, and the measurements were performed at an overpotential of −0.8 V in a 0.5 M H_2_SO_4_ solution.

Turnover frequencies were calculated from current densities (*j*) and number of active sites (Table S2) using the following equation:^35^
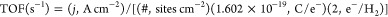
2

### 4.5 Finite Element Simulation of Electrostatics

Electrostatic simulations were conducted by using Comsol Multiphysics 5.2. The Poisson equation was numerically solved, assuming a potential difference between three 1 nm thick layers with micrometer width. Graphene was employed as the middle layer of the capacitor, and the whole structure was surrounded by water. The dielectric constants were extracted from the literature.

### 4.6 DFT Calculation Methods

All calculations were carried out using the atomistic simulation software QuantumATK (QuantumATK version T-2022.03)^37^ using a numerical LCAO basis set. The generalized gradient approximation (GGA) with the Perdew–Burke–Ernzerhof (PBE) functional was used to treat the exchange–correlation interactions in all calculations.^38^ The convergence thresholds for fluorographene, graphene, and MoS_2_ were set to 0.05 eV Å^–1^ for the atomic forces, and the tolerance accuracy of the self-consistent-field (SCF) loop was set to 0.0027 eV. To simulate the heterojunction nanoribbon, the 3D periodic boundary contained at least 15 Å of vacuum space to prevent the interaction between layers and nanoribbons.

In our calculations, we constructed and optimized fluorographene, graphene, and MoS_2_, respectively, and constructed a heterostructure. For the heterojunction nanoribbon model, the top layer is a one-side-saturated fluorinated graphene, and the second layer is pure graphene with optimization. To minimize the effect of lattice mismatch between graphene and MoS_2_, we enlarged the unit cell of graphene and MoS_2_ by 4 times and 3 times to build a heterojunction nanoribbon structure (Figures 4a,b and S6).^39^ To further understand the interaction and energy between hydrogen and nanoribbon, a hydrogen atom was placed adjacent to the basal plane and edges of layers individually, and the total energy was calculated for each model. Finally, following the methodology outlined in Nørskov et al.,^40^ the Gibbs free energy of the H atom was obtained.

The total energy (Δ*E*_H_*) and the Gibbs free energy (Δ*G*_H_*) are calculated as
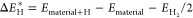
3
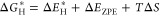
4*E*_material + H_ and *E*_material_ are the total energy of the given unit cell with and without atomic hydrogen in a vacuum from simulation. *E*_H_2__ is the energy of one hydrogen in the gas phase. Δ*E*_H_* is the binding energy of atomic hydrogen on the given unit cell. Δ*E*_ZPE_ is the zero-point energy difference between the adsorbed hydrogen and hydrogen in the gas phase, and Δ*S* is one hydrogen entropy between the absorbed state and gas phase, which can be calculated as −1/2 *S*_0_ (*S*_0_ is the entropy of H_2_ in the 5 gas phase at standard conditions, 1 bar of H_2_, and pH = 0 at 300 K). Considering all of the previously mentioned factors, Δ*G*_H_* = Δ*E*_H_* + 0.24 eV.

## Supporting Information Available

In-depth characterization of material and nanoribbon formation, description of electrochemical data processing, extended simulation results, and comparison of Tafel slope and turnover frequency (TOF) to previous results. (PDF)

## Abbreviations

OEFs: oriented electric fields
HER: hydrogen evolution reaction
SLG: single-layer graphene
TLG: trilayer graphene
TOF: turnover frequency
