# Supplementary material for: Enhancing the Electrochemical Activity of 2D Materials Edges through Oriented Electric Fields
Source: ACS Nano. 2024 Jul 16;18(30):19828–35. doi: 10.1021/acsnano.4c06341 (PMC11295188; doi:10.1021/acsnano.4c06341)
Supplement: Supplementary file 1 — nn4c06341_si_001.pdf [file nn4c06341_si_001.pdf]

Supporting Information for
Enhancing the Electrochemical Activity of 2D Materials Edges through Oriented Electric Fields

Hao Wang[a,b#], Ding-Rui Chen[a,c,d#], You-Chen Lin[a], Po-Han Lin[b], Jui-Teng Chang[b], Jeyavelan Muthu[e], Mario Hofmann[b], Ya-Ping Hsieh[a]*

[a] *Institute of Atomic and Molecular Sciences, Academia Sinica, Taipei, 10617, Taiwan*

[b] *Department of Physics, National Taiwan University, Taipei, 10617, Taiwan*

[c]*International Graduate Program of Molecular Science and Technology, National Taiwan University, Taipei, 10617, Taiwan*

[d]*Molecular Science and Technology Program, Taiwan International Graduate Program, Academia Sinica, Taipei, 10617, Taiwan*

[e] *J. Heyrovský Institute of Physical Chemistry, Department of Low Dimensional Systems, Prague, Czech Republic*

*Corresponding author Email: yphsieh@gate.sinica.edu.tw

Supplementary Figures

Detailed Characterization of the etching process

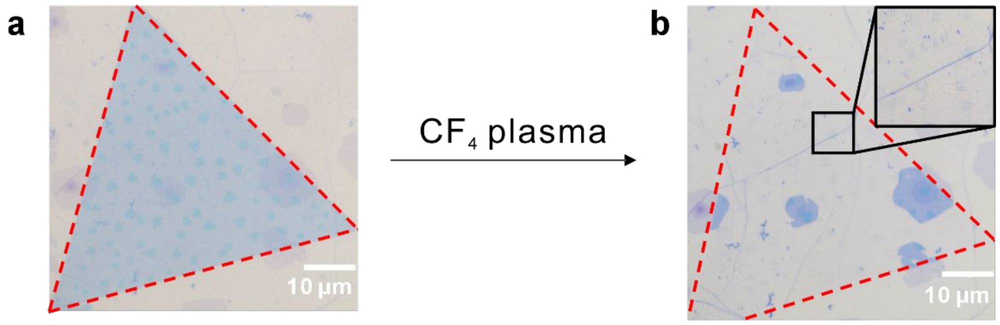

Figure S1. (a,b) Optical micrograph of graphene-covered MoS_2 flake before/after CF_4 plasma treatment.

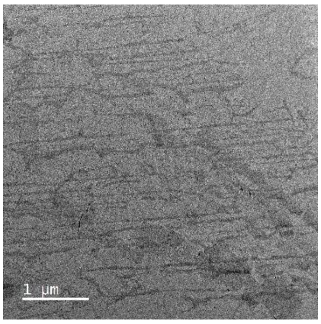

Figure S2. Transmission electron images of nanoribbon heterojunction.

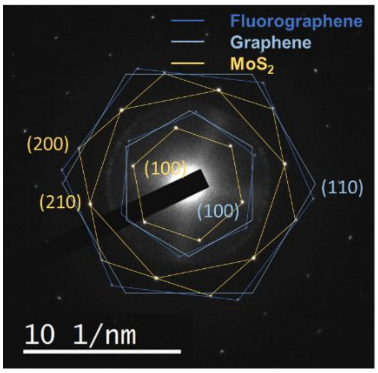

Figure S3. Selected-area electron diffraction (SAED) shows three sets of hexagonal diffraction patterns. One set was assigned to the (100), (110) and (200) MoS_2 planes with a lattice spacing of 0.273 nm, 0.160 nm, and 0.137 nm respectively, agreeing with previous reports.[1] The other sets correspond to the (100) and (110) planes of the graphene and fluorographene lattice. The extracted lattice spacing of 0.213nm and 0.123 nm agrees with both lattice types.[2]

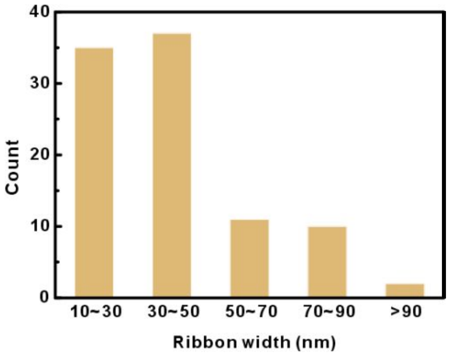

Figure S4. Histogram of heterostructure nanoribbon width measured from transmission electron images

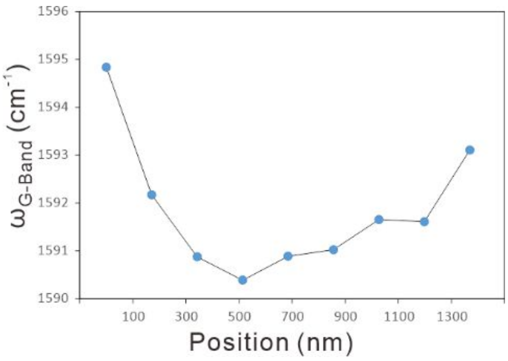

Figure S5. Raman peak shift of graphene G-band across the nanoribbon as indicated in Figure 2c

Description of electrochemical data processing

The current density within the nanoribbon array was calculated according to

$$J = \frac{n_{NR} A_{NR}}{A_{window}} I$$

, where n_{NR} is the number density of nanoribbons, A_{NR} is the average area of one nanoribbon, and A_{window} is the area of the exposed microelectrode. The parameters for n_{NR} and A_{NR} are extracted from statistical analysis of atomic force micrographs and transmission electron micrographs and we utilize a nanoribbon width of 100nm and a length of 10um.

The extracted current density does not consider selective reactions at the edges and thus only provides a lower boundary for the reaction current. Despite this limitation, the extracted current density is significantly enhanced, demonstrating the impact of the increased edge reactivity. Previous work has suggested a hundredfold increased activity of edges compared to the basal plane[3], indicating that the current density for exclusive edge-based HER would be 30x higher.

To calculate the turnover frequency, we again utilize a conservative estimation procedure. We normalize the reaction current by both MoS_2 edge atoms and F-atoms. To calculate the number of edge-bound Mo atoms, we divide the perimeter of a nanoribbon by the MoS_2 lattice constant, while the F-atom density was derived to be 25% of the basal plane carbon atom concentration based on XPS characterization. This approach assumes identical turnover frequencies for F-atoms and MoS_2 edges, which provides a lower estimate of the TOF. Previous reports demonstrated the limited HER activity of fluorographene[4], which suggests that the real TOF of MoS_2 edges could be up to 150x higher.

The LSV curves with and without IR correction are shown below, and the iR corrected LSV curves were used to analyze the Tafel slope in Figure 5b.

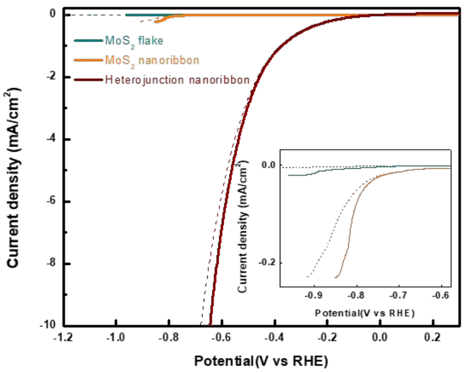

Figure. S6. Polarograms showing original (solid line) and iR corrected LSV curves, (inset) closeup of MoS2 flake and nanoribbon

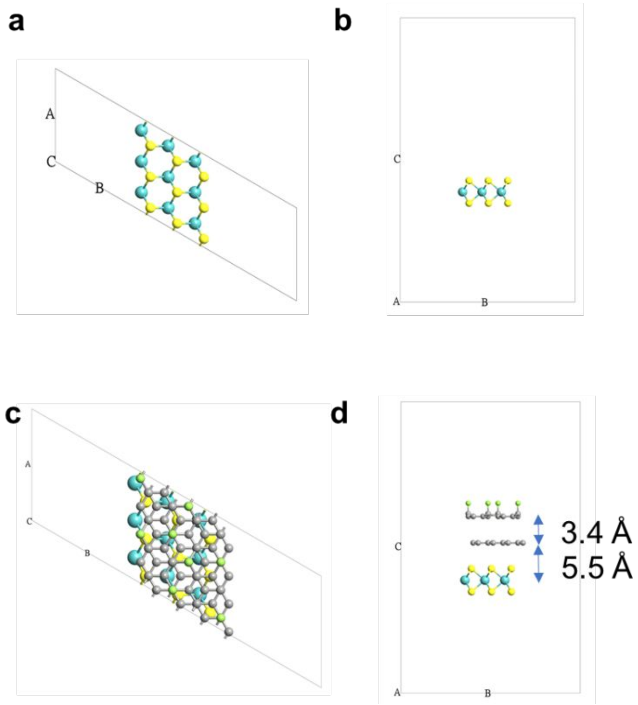

Figure. S7. Model of (a, b) MoS_2 nanoribbon and (c, d) heterostructure nanoribbon. The dimensions of both unit cell are fixed at 0.948 nm (A axis), 2.94 nm (B axis), and 4.00 nm (C axis). Elements: blue, Mo; yellow, S; green, F

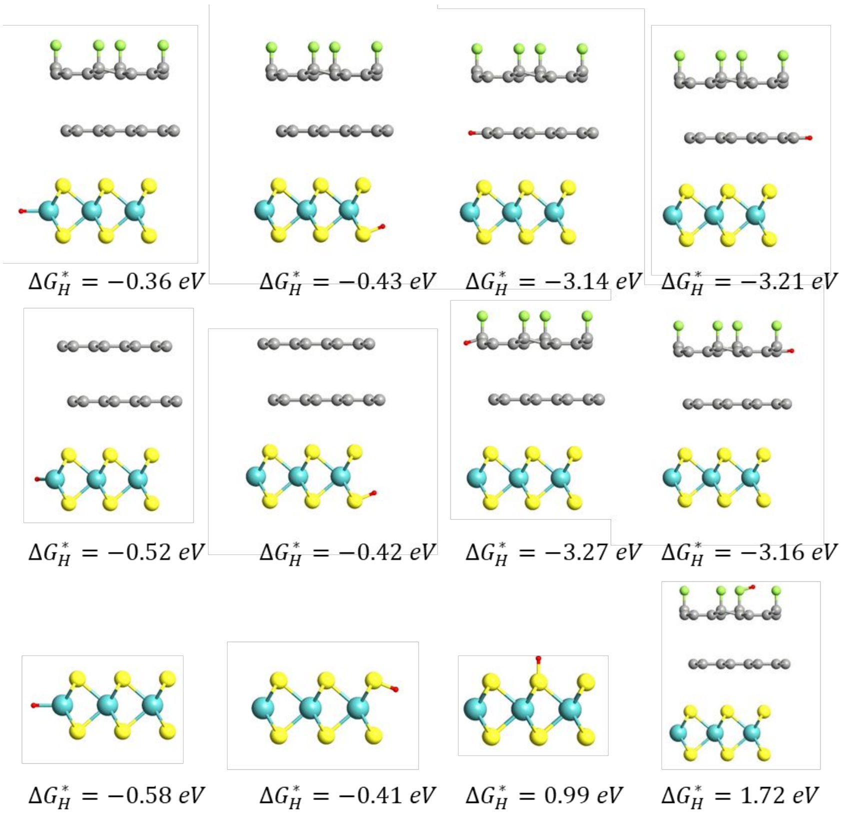

Figure S8. ΔG_H^* at different adsorption sites of MoS_2 nanoribbon and heterostructure nanoribbon. Elements: blue, Mo; yellow, S; green, F; red, H.

Supplementary Tables

Table T1. EIS fitting data: electrolyte resistance (R_s), parallel charge transfer resistance (R_{ct}), constant phase element (CPE)

	MoS_2 flake	MoS_2 nanoribbon	Heterojunction Nanoribbon
$R_s(\Omega)$	151.6	1737	335.6
$R_{ct}(\Omega)$	1.23×10^{7}	1.21×10^{6}	18900
CPE-T	7.15×10^{-11}	4.05×10^{-11}	1.38×10^{-7}
CPE-P	0.96	1.00	0.99

Table T2. number of active sites cm^{-2}

	MoS_2 flake	MoS_2 nanoribbon	Heterojunction Nanoribbon
# of active sites cm^{-2}	1.50×10^{14}	6.72×10^{13}	3.35×10^{14}

Table T3. Comparison of Tafel slope and turnover frequency (TOF) for our work with references

Materials type	Tafel slope (mV dec^{-1})	TOF (s^{-1})	Current density @-200mV (mA cm^{-2})	Ref.
**pristine MoS_2**	**221**	**0.52**	**3.5×10^{-4}**	**Our work**
pristine MoS_2	120	0.013	0.096	[5]
pristine MoS_2	61	0.02	>100	[6]
pristine MoS_2	121	0.096	6.8	[7]
pristine MoS_2	74	0.11	53	[8]
pristine MoS_2	153	3.00×10^{-3}	8.37×10^{-4}	[9]
pristine MoS_2	160	0.617	0.6	[10]
pristine MoS_2	155	0.7	4.46	[11]
**pristine MoS_2 (edge)**	**90**	**2.41**	**1.4×10^{-3}**	**Our work**
**OEF MoS_2 edge**	**69**	**7.23**	**0.551**	**Our Work**
pristine MoS_2 (edge)	160	0.1	>10	[12]
pristine MoS_2 (edge)	69	0.66	52.9	[7]
pristine MoS_2 (edge)	94	0.78	1.3×10^{-3}	[9]
pristine MoS_2 (edge)	59	1.51	152.9	[7]
pristine MoS_2 (edge)	60	1.64×10^{-2}	>0.3	[13]
pristine MoS_2 (edge)	200	3	6×10^{-3}	[14]
pristine MoS_2 (edge)	75	7.5	12.4	[15]
other catalyst (Hg)	-	1.04×10^{-9}	-	[13]
other catalyst (2H-WS_2)	-	0.078	5	[16]
other catalyst (Mo_2C)	63	-	56	[6]

other catalyst (MoSe)	118	0.014	0.11	[5]
other catalyst (MoP)	50	0.024	>120	[17]
other catalyst (Pt)	87	0.32	>40	[18]
other catalyst (Ru)	107	0.04	>40	[18]
other catalyst (c-$RuSe_2$)	130	0.07	40	[18]
other catalyst ($[Mo_3S_4]^{4+}$)	120	0.07	5~10	[19]
other catalyst (Fe-CoS_2)	73	0.17	>60	[20]
other catalyst (CoS_2)	90	0.17	>60	[20]
other catalyst (Pt/C)	30	0.28	>60	[21]
other catalyst (h-$RuSe_2$)	95	0.34	>40	[18]
other catalyst (P-CoS_2)	57	0.55	>60	[20]
other catalyst (C-Ir NSs)	36	0.6	>90	[22]
other catalyst (Fe/P-CoS_2)	56	1.33	>60	[20]
other catalyst (AC-Ir NS)	27	3.6	>90	[22]
other catalyst (Ru/NC)	17	8.9	>60	[21]
defected/functionalized MoS_2	82	0.05	3	[23]
defected/functionalized MoS_2	60	0.08	16	[23]
defected/functionalized MoS_2	62	0.1	200	[24]
defected/functionalized MoS_2	60	0.3	13	[25]
defected/functionalized MoS_2	40	0.5	5.97	[26]
defected/functionalized MoS_2	120	0.88	-	[10]
defected/functionalized MoS_2	133	1	0.32	[10]
defected/functionalized MoS_2	130	1.11	0.6	[10]
defected/functionalized MoS_2	118	1.14	0.6	[10]
defected/functionalized MoS_2	117	1.19	0.6	[10]

defected/functionalized MoS_2	105	1.3	0.65	[10]
defected/functionalized MoS_2	108	1.32	0.6	[10]
defected/functionalized MoS_2	85	2	1.19	[27]
defected/functionalized MoS_2	193	2	0.38	[28]
defected/functionalized MoS_2	85	3.2	-	[12]
defected/functionalized MoS_2	136	8	0.039	[14]
combination of materials	48	0.17	>100	[6]
combination of materials	47	1.45	0.1~1	[29]
combination of materials	40	3.5	>25	[30]
combination of materials	60	4	1.05	[31]
combination of materials	50	0.12	>120	[17]

References

(1) Li, B.; Yang, S.; Huo, N.; Li, Y.; Yang, J.; Li, R.; Fan, C.; Lu, F. Growth of large area few-layer or monolayer MoS_2 from controllable MoO_3 nanowire nuclei. *Rsc Advances* **2014**, *4* (50), 26407-26412. Xu, X.; Liu, L. MoS_2 with controlled thickness for electrocatalytic hydrogen evolution. *Nanoscale Research Letters* **2021**, *16* (1), 137.
(2) Robertson, A. W.; Warner, J. H. Hexagonal single crystal domains of few-layer graphene on copper foils. *Nano letters* **2011**, *11* (3), 1182-1189.
(3) Raman, R.; Muthu, J.; Yen, Z.-L.; Qorbani, M.; Chen, Y.-X.; Chen, D.-R.; Hofmann, M.; Hsieh, Y.-P. Selective activation of MoS_2 grain boundaries for enhanced electrochemical activity. *Nanoscale Horizons* **2024**, *9* (6), 946-955.
(4) Seydou, M.; Lassoued, K.; Tielens, F.; Maurel, F.; Raouafi, F.; Diawara, B. A DFT-D study of hydrogen adsorption on functionalized graphene. *RSC Advances* **2015**, *5* (19), 14400-14406.
(5) Kong, D.; Wang, H.; Cha, J. J.; Pasta, M.; Koski, K. J.; Yao, J.; Cui, Y. Synthesis of MoS_2 and $MoSe_2$ films with vertically aligned layers. *Nano letters* **2013**, *13* (3), 1341-1347.
(6) Zhao, Z.; Qin, F.; Kasiraju, S.; Xie, L.; Alam, M. K.; Chen, S.; Wang, D.; Ren, Z.; Wang, Z.; Grabow, L. C. Vertically aligned MoS_2/Mo_2C hybrid nanosheets grown on

carbon paper for efficient electrocatalytic hydrogen evolution. *ACS Catalysis* **2017**, *7* (10), 7312-7318.
(7) Hu, J.; Huang, B.; Zhang, C.; Wang, Z.; An, Y.; Zhou, D.; Lin, H.; Leung, M. K.; Yang, S. Engineering stepped edge surface structures of MoS_2 sheet stacks to accelerate the hydrogen evolution reaction. *Energy & Environmental Science* **2017**, *10* (2), 593-603.
(8) Guo, B.; Yu, K.; Li, H.; Song, H.; Zhang, Y.; Lei, X.; Fu, H.; Tan, Y.; Zhu, Z. Hollow structured micro/nano MoS_2 spheres for high electrocatalytic activity hydrogen evolution reaction. *ACS applied materials & interfaces* **2016**, *8* (8), 5517-5525.
(9) Chen, D.-R.; Muthu, J.; Guo, X.-Y.; Chin, H.-T.; Lin, Y.-C.; Haider, G.; Ting, C.-C.; Kalbáč, M.; Hofmann, M.; Hsieh, Y.-P. Edge-dominated hydrogen evolution reactions in ultra-narrow MoS_2 nanoribbon arrays. *Journal of Materials Chemistry A* **2023**, *11* (29), 15802-15810.
(10) Tao, L.; Duan, X.; Wang, C.; Duan, X.; Wang, S. Plasma-engineered MoS_2 thin-film as an efficient electrocatalyst for hydrogen evolution reaction. *Chemical communications* **2015**, *51* (35), 7470-7473.
(11) Wang, Y.; Udyavara, S.; Neurock, M.; Frisbie, C. D. Field effect modulation of electrocatalytic hydrogen evolution at back-gated two-dimensional MoS_2 electrodes. *Nano letters* **2019**, *19* (9), 6118-6123.
(12) Guoqing, L.; Du, Z.; Qiao, Q.; Yifei, Y.; David, P.; Abdullah, Z.; Raj, K.; Stefano, C.; Frank, H.; Steve, S. All The Catalytic Active Sites of MoS_2 for Hydrogen Evolution. **2016**.
(13) Conway, B.; Tilak, B. Interfacial processes involving electrocatalytic evolution and oxidation of H_2, and the role of chemisorbed H. *Electrochimica acta* **2002**, *47* (22-23), 3571-3594.
(14) Zhang, R.; Zhang, M.; Yang, H.; Li, G.; Xing, S.; Li, M.; Xu, Y.; Zhang, Q.; Hu, S.; Liao, H. Creating fluorine ‐ doped MoS_2 edge electrodes with enhanced hydrogen evolution activity. *Small Methods* **2021**, *5* (11), 2100612.
(15) Li, G.; Zhang, D.; Qiao, Q.; Yu, Y.; Peterson, D.; Zafar, A.; Kumar, R.; Curtarolo, S.; Hunte, F.; Shannon, S. All the catalytic active sites of MoS_2 for hydrogen evolution. *Journal of the American Chemical Society* **2016**, *138* (51), 16632-16638.
(16) Xie, L.; Wang, L.; Zhao, W.; Liu, S.; Huang, W.; Zhao, Q. WS_2 moiré superlattices derived from mechanical flexibility for hydrogen evolution reaction. *Nature communications* **2021**, *12* (1), 5070.

(17) Kibsgaard, J.; Jaramillo, T. F. Molybdenum phosphosulfide: an active, acid‐stable, earth‐abundant catalyst for the hydrogen evolution reaction. *Angewandte Chemie International Edition* **2014**, *53* (52), 14433-14437.
(18) Zhao, Y.; Cong, H.; Li, P.; Wu, D.; Chen, S.; Luo, W. Hexagonal $RuSe_2$ nanosheets for highly efficient hydrogen evolution electrocatalysis. *Angewandte Chemie* **2021**, *133* (13), 7089-7093.
(19) Jaramillo, T. F.; Bonde, J.; Zhang, J.; Ooi, B.-L.; Andersson, K.; Ulstrup, J.; Chorkendorff, I. Hydrogen evolution on supported incomplete cubane-type $[Mo_3S_4]^{4+}$ electrocatalysts. *The Journal of Physical Chemistry C* **2008**, *112* (45), 17492-17498.
(20) Zhang, Y.-Y.; Zhang, X.; Wu, Z.-Y.; Zhang, B.-B.; Zhang, Y.; Jiang, W.-J.; Yang, Y.-G.; Kong, Q.-H.; Hu, J.-S. Fe/P dual doping boosts the activity and durability of CoS_2 polycrystalline nanowires for hydrogen evolution. *Journal of materials chemistry A* **2019**, *7* (10), 5195-5200.
(21) Li, Y.; Liu, H.; Li, B.; Yang, Z.; Guo, Z.; He, J.-B.; Xie, J.; Lau, T.-C. Ru single atoms and nanoclusters on highly porous N-doped carbon as a hydrogen evolution catalyst in alkaline solutions with ultrahigh mass activity and turnover frequency. *Journal of Materials Chemistry A* **2021**, *9* (20), 12196-12202.
(22) Wu, G.; Han, X.; Cai, J.; Yin, P.; Cui, P.; Zheng, X.; Li, H.; Chen, C.; Wang, G.; Hong, X. In-plane strain engineering in ultrathin noble metal nanosheets boosts the intrinsic electrocatalytic hydrogen evolution activity. *Nature Communications* **2022**, *13* (1), 4200.
(23) Li, H.; Tsai, C.; Koh, A. L.; Cai, L.; Contryman, A. W.; Fragapane, A. H.; Zhao, J.; Han, H. S.; Manoharan, H. C.; Abild-Pedersen, F. Activating and optimizing MoS_2 basal planes for hydrogen evolution through the formation of strained sulphur vacancies. *Nature materials* **2016**, *15* (1), 48-53.
(24) Wang, H.; Lu, Z.; Kong, D.; Sun, J.; Hymel, T. M.; Cui, Y. Electrochemical tuning of MoS_2 nanoparticles on three-dimensional substrate for efficient hydrogen evolution. *ACS nano* **2014**, *8* (5), 4940-4947.
(25) Benck, J. D.; Chen, Z.; Kuritzky, L. Y.; Forman, A. J.; Jaramillo, T. F. Amorphous molybdenum sulfide catalysts for electrochemical hydrogen production: insights into the origin of their catalytic activity. *Acs Catalysis* **2012**, *2* (9), 1916-1923.
(26) Yin, Y.; Han, J.; Zhang, Y.; Zhang, X.; Xu, P.; Yuan, Q.; Samad, L.; Wang, X.; Wang, Y.; Zhang, Z. Contributions of phase, sulfur vacancies, and edges to the hydrogen evolution reaction catalytic activity of porous molybdenum disulfide nanosheets. *Journal of the American Chemical Society* **2016**, *138* (25), 7965-7972.
(27) Li, L.; Qin, Z.; Ries, L.; Hong, S.; Michel, T.; Yang, J.; Salameh, C.; Bechelany, M.; Miele, P.; Kaplan, D. Role of sulfur vacancies and undercoordinated Mo regions

in MoS_2 nanosheets toward the evolution of hydrogen. *ACS nano* **2019**, *13* (6), 6824-6834.
(28) Tsai, C.; Li, H.; Park, S.; Park, J.; Han, H.; Nørskov, J.; Zheng, X.; Abild-Pedersen, F. Electrochemical generation of sulfur vacancies in the basal plane of MoS_2 for hydrogen evolution. Nat. Commun. 8, 15113. 2017.
(29) Seokhee, S.; Zhenyu, J.; Ranjith, B.; Yo-Sep, M. High Turnover Frequency of Hydrogen Evolution Reaction on Amorphous MoS_2 Thin Film Directly Grown by Atomic Layer Deposition. **2015**.
(30) Li, D. J.; Maiti, U. N.; Lim, J.; Choi, D. S.; Lee, W. J.; Oh, Y.; Lee, G. Y.; Kim, S. O. Molybdenum sulfide/N-doped CNT forest hybrid catalysts for high-performance hydrogen evolution reaction. *Nano letters* **2014**, *14* (3), 1228-1233.
(31) Chen, Z.; Cummins, D.; Reinecke, B. N.; Clark, E.; Sunkara, M. K.; Jaramillo, T. F. Core–shell MoO_3–MoS_2 nanowires for hydrogen evolution: a functional design for electrocatalytic materials. *Nano letters* **2011**, *11* (10), 4168-4175.
